# Serotype-specific detection of dengue viruses in a nonstructural protein 1-based enzyme-linked immunosorbent assay validated with a multi-national cohort

**DOI:** 10.1371/journal.pntd.0008203

**Published:** 2020-06-24

**Authors:** Irene Bosch, Ankita Reddy, Helena de Puig, Juan E. Ludert, Federico Perdomo-Celis, Carlos F. Narváez, Alice Versiani, Diana Fandos, Mauricio L. Nogueira, Mohit Singla, Rakesh Lodha, Guruprasad R. Medigeshi, Ivette Lorenzana, Hugo Vicente Ralde, Margarita Gélvez-Ramírez, Luis A. Villar, Megan Hiley, Laura Mendoza, Nol Salcedo, Bobby Brooke Herrera, Lee Gehrke

**Affiliations:** 1 E25Bio, Cambridge, Massachusetts, United States of America; 2 Institute for Medical Engineering & Science, Massachusetts Institute of Technology, Cambridge, Massachusetts, United States of America; 3 Department of Medicine, Mount Sinai School of Medicine, New York, New York, United States of America; 4 Wyss Institute for Biologically Inspired Engineering, Harvard Medical School, Boston, Massachusetts, United States of America; 5 Departamento de Infectómica y Patogénesis Molecular, Centro de Investigación y de Estudios Avanzados del Instituto Politécnico Nacional (CINVESTAV-IPN), Ciudad de México, México; 6 Programa de Medicina, Facultad de Salud, Universidad Surcolombiana, Neiva, Colombia; 7 Department of Infectious and Parasitic Diseases, Faculdade de Medicina de São José do Rio Preto, SP, Brazil; 8 Institut Químic de Sarrià, Universitat Ramon Llull, Barcelona, Spain; 9 Faculdade de Medicina de São José do Rio Preto (FAMERP), São José do Rio Preto, Brazil; 10 Department of Paediatrics, All India Institute of Medical Sciences, Ansari Nagar, New Delhi, India; 11 Translational Health Science and Technology Institute, Faridabad, India; 12 Instituto de Investigación en Microbiología, Universidad Nacional Autónoma de Honduras, Tegucigalpa, Honduras; 13 Facultad de Medicina, Universidad Autónoma de Guadalajara, Guadalajara, Mexico; 14 Universidad Industrial de Santander and AEDES Network, Bucaramanga, Santander, Colombia; 15 Department of Immunology and Infectious Diseases, Harvard T.H. Chan School of Public Health, Boston, Massachusetts, United States of America; 16 Department of Microbiology and Immunobiology, Harvard Medical School, Boston, Massachusetts, United States of America; University of Geneva Hospitals, SWITZERLAND

## Abstract

**Background:**

Dengue virus (DENV) infections pose one of the largest global barriers to human health. The four serotypes (DENV 1–4) present different symptoms and influence immune response to subsequent DENV infections, rendering surveillance, risk assessments, and disease control particularly challenging. Early diagnosis and appropriate clinical management is critical and can be achieved by detecting DENV nonstructural protein 1 (NS1) in serum during the acute phase. However, few NS1-based tests have been developed that are capable of differentiating DENV serotypes and none are currently commercially available.

**Methodology/Principle findings:**

We developed an enzyme-linked immunosorbent assay (ELISA) to distinguish DENV-1-4 NS1 using serotype-specific pairs of monoclonal antibodies. A total of 1,046 antibodies were harvested from DENV-immunized mice and screened for antigen binding affinity. ELISA clinical performance was evaluated using 408 polymerase chain reaction-confirmed dengue samples obtained from patients in Brazil, Honduras, and India. The overall sensitivity of the test for pan-DENV was 79.66% (325/408), and the sensitivities for DENV-1-4 serotyping were 79.1% (38/48), 80.41% (78/97), 100% (45/45), and 79.6% (98/123), respectively. Specificity reached 94.07–100%.

**Significance:**

Our study demonstrates a robust antibody screening strategy that enabled the development of a serotype NS1-based ELISA with maximized specific and sensitive antigen binding. This sensitive and specific assay also utilized the most expansive cohort to date, and of which about half are from Latin America, a geographic region severely underrepresented in previous similar studies. This ELISA test offers potential enhanced diagnostics during the acute phase of infection to help guide patient care and disease control. These results indicate that this ELISA is a promising aid in early DENV-1-4 diagnosis and surveillance in regions of endemicity in addition to offer convenient monitoring for future vaccine interventions.

## Author summary

Dengue virus (DENV) infection is an increasingly significant threat to global health, with a yearly estimate of 390 million infections and an expected increasing burden with the rise of climate change and globalization. DENV is caused by one of the four serotypes (DENV-1-4), each of which have been associated with different immune responses and clinical manifestations. We developed a method to detect DENV serotypes by targeting the nonstructural 1 (NS1) antigen through an enzyme-linked immunosorbent-based assay (ELISA) with high sensitivity and specificity. We demonstrate that our high throughput mouse-derived antibody screening method selected for optimal test performance. The antibodies were integrated into an ELISA that can distinguish between the four different dengue serotypes by serotype-specific pairing. In addition, we provide a dengue universal antibody combination that enables pan-virus detection independently of the serotype. We use the ELISA in three different countries and calculate overall and site-specific sensitivities and specificities. The assay performs optimally when levels of viremia are high during the first five days of fever.

**Key points**

- A Dengue virus serotype-specific non-structural protein 1 (NS1)-based ELISA was developed with high sensitivity and specificity
- Evaluation using a large multinational cohort highlights the potential for commercial use

## Introduction

Dengue virus (DENV) is currently the most significant arthropod-borne virus (arbovirus), endemic in tropical and subtropical countries, with a yearly estimate of 390 million infections, of which 96 million are symptomatic [1–3]. The widespread distribution of the principal vector, *Aedes aegypti*, makes this a global health concern as around half of the world’s population is at risk of contracting the disease [3]. As the rate of climate change, urbanization, globalization, vector distribution, and population levels continue to spread, DENV infections are expected to pose an even greater threat to global health [4, 5]. In addition, international travel is increasingly a contributing factor as travelers often import dengue or fall sick [6–8]. Worldwide initiatives implemented to prevent the infection have not yet been successful at eradicating the disease.

The treatment for dengue fever is mainly supportive, but failure to adequately identify and treat patients can result in complications that can be fatal [1, 3]. Many febrile illnesses such as Zika and Chikungunya present similar symptoms and are spread by the same mosquito vector, commonly resulting in difficult clinical differential diagnosis [9]. In light of the recent development of the dengue vaccine, proper medical documentation of the infecting DENV serotypes would be ideal, given that the vaccines currently in clinical trials have been recommended in those individuals who have experienced a primary dengue infection. This usage limitation may help reduce specific vaccine complications [10]. Timely identification of DENV serotypes can aid in the triaging and managing of patients, enabling rapid clinical response and appropriate epidemiological surveillance of diseases during outbreaks [1].

Dengue is caused by one of four DENV serotypes (DENV-1-4), and these serotypes have been associated with varying symptoms [11–13]. Once recovery occurs from a DENV infection by any given serotype, the patient gains permanent homologous and transient heterologous immunity. In humans, if a subsequent heterologous infection occurs, the risk of developing severe dengue increases; these results were corroborated in a recent study demonstrating that in a narrow range of preexisting anti-DENV antibodies, the hazard of severe disease was significantly increased [2, 3, 14, 15].

Infections by DENV-2 have been more strongly associated in previous studies with severe dengue disease (SDD), including dengue hemorrhagic fever (DHF) and dengue shock syndrome (DSS), regardless of age or gender [16–18]. DENV-3 has also been associated with severe forms of dengue, including shock and severe liver involvement [18, 19]. As for DENV-1 and DENV-4, less severe clinical manifestations have been linked to these two serotypes, with DENV-4 viral titers being the lowest amongst the other serotypes [18, 20].

Methods of diagnosis currently include nucleic acid detection through polymerase chain reaction (PCR), IgG/IgM detection through enzyme-linked immunosorbent assays (ELISAs) and lateral flow tests [21]. These procedures have many costs and technical limitations. IgG/IgM-based tests to DENV envelope have reported cross-reactive properties against other flaviviruses and have a detection window that prevents acute phase diagnosis (>5 days) [22–24]. During dengue infection, the less conserved non-structural protein 1 (NS1) is secreted in high concentrations into the blood. NS1 levels can remain in the bloodstream for 9–18 days, rendering NS1 an ideal target for disease detection [25, 26]. Two previous studies have reported varying results for DENV-1-4 serotype detection on a small subset of samples, primarily focusing on Asian populations [27, 28]. Additionally, serological and cellular assays were recently evaluated to determine prior dengue infections [29–32]. Bosch et al. demonstrated that rapid antigen lateral flow test differentially detects the four serotypes of dengue through monoclonal antibody (mAb) pairing without displaying cross-reactivity to Zika virus (ZIKV) [33]. In this study, the performance of ELISA assays for the detection of pan-DENV and the four serotypes are reported. The sensitivity and specificity of the tests enable an early and accurate diagnosis that can improve health responses in areas in which DENV-1-4 co-circulate.

## Materials and methods

### Study design

The overall objective of the study was to develop a serotype specific NS1-based ELISA, and to validate the sensitivity and specificity of the test in endemic areas, comparing them to PCR diagnosis. All tests were performed on-site, among them the University of Tegucigalpa in Honduras, Faculdade de Medicina de Sao Jose do Rio Preto, the Federal University of São João del-Rei in Brazil, and the Translational Health Science and Technology Institute in India.

### Clinical samples

In total, 408 RT-PCR-serotyped dengue samples from Brazil, India, and Honduras, along with 17 negative patient samples were included in the study. The geographic breakdown was as follows: 300 positive and 2 negative patient samples in Brazil, 65 positive and 2 negative patient samples in India, and 43 positive and 13 negative patient samples in Honduras. In our ELISAs, we used 1,228 negative wells which were from patients who tested negative for DENV and patients who tested negative for the serotype of interest. All clinical serum samples were de-identified and collected during the acute phase (1 to 5 days after the onset of illness). Samples were collected and processed for RNA extraction followed by RT-PCR in each of the participating laboratories. Twenty five samples from Colombia and 25 samples from Mexico were used to optimize the methodology. All samples were collected prior to ZIKV introduction in Latin America.

### Ethics statement

All patients from each of the cohorts provided informed consent for the original collection of the de-identified samples. The primary studies under which the samples and data were collected received an exemption determination from the Massachusetts Institute of Technology Review Board (IRB) and local research ethics committees at University of Tegucigalpa, Honduras, Federal University of São João del-Rei in Brazil, and the Translational Health Science and Technology Institute in India.

### Antibody selection

Four C57BL/6 mice, HM7729, HM7732, MA724, and MA725, were immunized with DENV-1, DENV-2, DENV-3, and DENV-4, respectively. From the four mice, a total of 1046 antibodies (1–1046) were harvested from hybridomas and their binding to DENV 1–4 NS1 recombinant antigens was measured by ELISA. The data showed positive reaction against each of the four serotypes. The top 50 antibodies that presented the highest OD_450_ using the primary ELISA screen when tested in a secondary screen by fluorescent-activated cell sorting (FACS) designed to evaluate the expression of native NS1 in infected Vero cells. The antibodies resulting positive with the FACS were isotyped and purified using Protein L or protein G and the pairing of antibodies was used in a dipstick format to obtain the pairs with lowest limit of detection and dissociation constant (K_d_) after image analysis of the intensity of the test band obtained.

### ELISA for the detection and quantification of DENV NS1

Detection antibodies were first biotinylated using Thermo Scientific EZ-Link Sulfo-NHS-LC-Biotinylation Kit. To prepare the ELISA, ninety-six-well plates (flat bottom high binding, CoStar; cat. No 3590) were coated with 100 μl of the specific antibody (PAN DENV: mAb 323; DENV 1: mAb 271; DENV 2: mAb 323 or mAb 243; DENV 3: mAb 55; DENV 4: mAb 55) at a 1 μg/ml, diluted in 1X PBS pH 7.4 (ref. 10010–023, Gibco). After incubating the plates overnight at room temperature, the antibody was discarded and each well was incubated for 2 hours at room temperature with 200 μl/well of 5% Blotto made from 5% nonfat dry milk (cat. No. sc-2325, Santa Cruz Biotechnology) and 0.05% Tween 20 (cat. No. p-1379, Sigma-Aldrich) diluted in PBS. After discarding the Blotto, 50 μl of serum sample diluted in 50 μl 2.5% Blotto in PBS were incubated in each well for 1 hour at room temperature. After washing the plates three times with 0.1% Tween 20 in PBS, 100 μl/well of biotin-labeled mAb (PAN DENV: mAbs 411, 626, 243, 271; DENV 1: mAb 912; DENV 2: mAb 243 or mAb 164; DENV 3: mAb 411; DENV 4: mAb 626) at 1 μg/ml was incubated for 1 hour at room temperature. The plates were washed four times with the 0.1% Tween 20 solution. One hundred μl/well of peroxidase-labeled streptavidin High Sensitivity (Thermo-Fisher Scientific, cat. No 21130) at 1:1000 dilution, diluted in 2.5% Botto, was added and incubated for 1 hour at room temperature. The plates were again washed four times with the 0.1% Tween 20 in PBS. Following the wash steps, 100 μl/well of tetramethylbenzidine single solution (cat. No 002023, Life Technologies) were pipetted into each well to develop the color reaction and stopped by the addition of 50 μl/well of 2M Sulfuric acid (cat. No. 8315–32, Ricca Chemical Company). The plates were read by a TriStar LB 941 spectrophotometer (berthold Technologies) at a wavelength of 450 nm.

### RNA extraction and non-quantitative PCR

RNA was extracted according to the QIAamp Viral RNA Mini Handbook for purification of viral RNA from plasma, serum, cell-free body fluids and culture supernatants (Qiagen, Hilden, Germany). Virus identity including serotypes was determined using non-quantitative PCR. The Fast Track Diagnostics Dengue/Chik real-time PCR protocol and reagents were used to process samples in India. The Center for Disease Control and prevention (CDC) DENV 1–4 Real-Time qPCR protocol and reagents were used to process samples in Brazil. Finally, the CDC Trioplex Real-Time PCR protocol and reagents were used to process samples in Honduras.

### ROC analysis

To report the performance of the ELISA, Receiver Operating Characteristic (ROC) curves were created using GraphPad Prism 8.0 software. The ROC curve presents test performance as True Positive Rate (% sensitivity) versus False Positive Rate (100%—% specificity). Optimal cutoff values, which maximize sensitivity and specificity, were calculated from the ROC curve using GraphPad Prism 8.0. The sensitivity is defined as the fraction of total confirmed positive samples that are true positives according to the test. The specificity is defined as the fraction of total confirmed negative samples that are true negatives according to the test. Serotype data was analyzed using samples with a positive PAN value using the cutoff value determined for PAN-Dengue. Confidence intervals (CI) using the Wilson/Brown method and Area Under Curve (AUC) were calculated for each serotype and PAN using GraphPad.

## Results

### Antibody selection for serotype NS1 ELISA

From the 1,046 antibodies harvested from four representative immunized mice of NS1 from each of the serotypes, the following antibody pairs were selected for their best limit of detection [33]. ELISA capture antibodies were: pan-DENV: mAb 323; DENV 1: mAb 271; DENV 2: mAb 243 (India and Honduras) and mAb 323 (Brazil); DENV 3: mAb 55; DENV 4: mAb 55. Detection antibodies were biotinylated and used in the ELISA: PAN DENV: mAbs 411, 626, 243, 271; DENV 1: mAb 912; DENV 2: mAb 164 in India and mAb 243 in Brazil and Honduras; DENV 3: mAb 411; DENV 4: mAb 626. This selection was based upon binding affinity and inter-serotype specificity (Supp. S1 and S2 Figs).

### Limits of detection

The limits of detection and dissociation constants of each of the antibody pairs for the detection of its respective dengue serotype were calculated by ELISA or dipstick and fitting the data using a Langmuir equation. The limits of detection were between 33.96–354.10 ng/ml (Supp. S1 Table, Supp. S3 Fig) which is within the range of NS1 concentration found in dengue patients.

### Aggregate performance of DENV NS1 ELISA for serotype and PAN diagnosis [Table 1 and S1A–S1E Fig]

**Fig 1:**
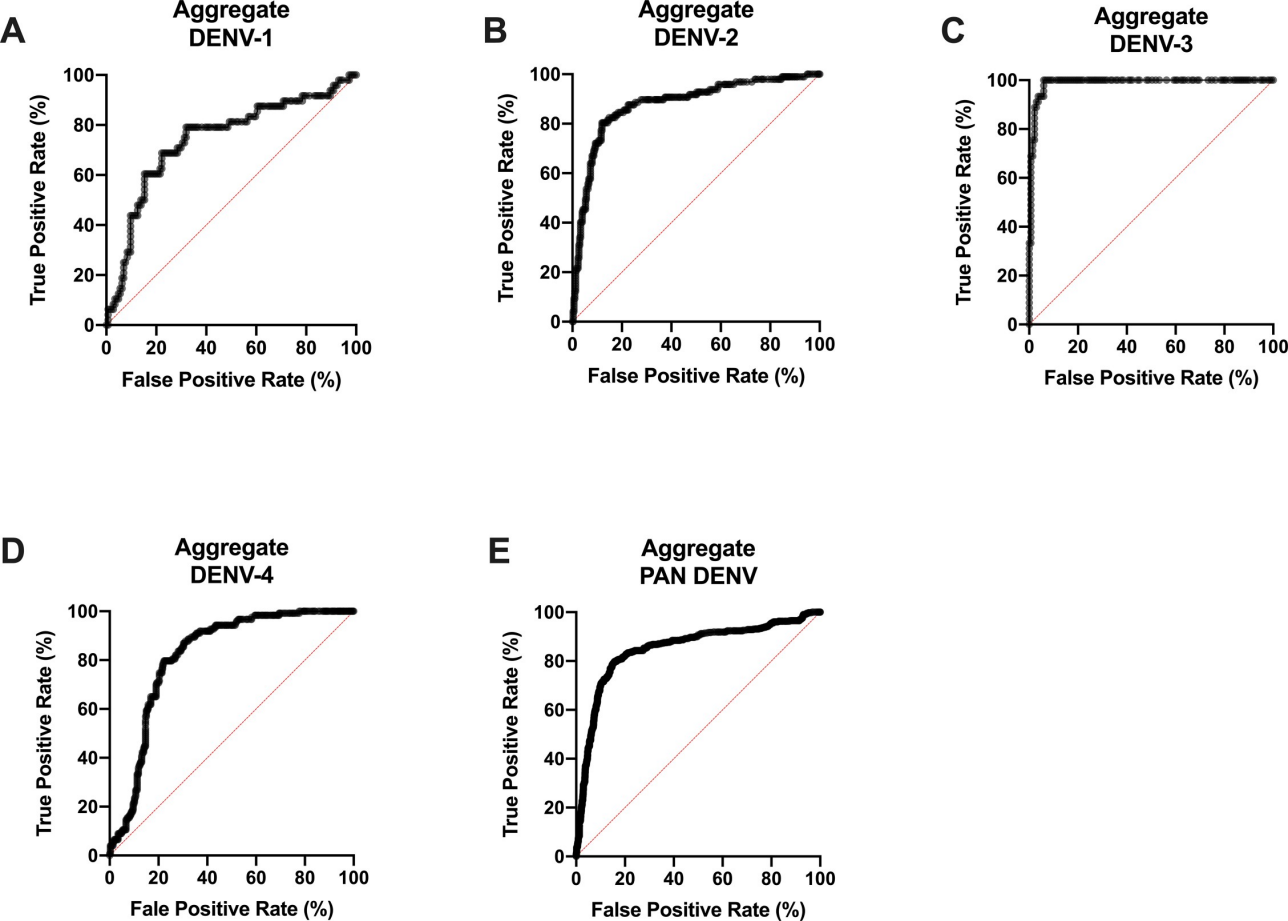
Performance of serotype DENV NS1 ELISA Test using aggregate samples. Receiver Operating Characteristic (ROC) Curve analysis of the aggregated patient sample data from Brazil, Honduras, and India collected for DENV-1 **(A)**, DENV-2 **(B)**, DENV-3 **(C)**, DENV-4 **(D)**, PAN DENV **(E)**. Performance is demonstrated in terms of True Positive Rate (%) versus False Positive Rate (%).

In total, 408 RT-PCR-serotyped dengue samples from Brazil, Honduras and India, along with 17 negative samples were tested with the serotype and pan-DENV NS1 ELISA. A total of 1,228 negative wells were used to calculate the intra-serotype specificity. The sensitivity and specificity of the developed NS1 serotype and pan-DENV ELISA were determined based upon the PCR-confirmed characterization (Table 1). As demonstrated by the area under the ROC curves (AUCs), which ranged from 0.74 to 0.99, the ELISA presented high sensitivity and specificity. Sensitivity is defined as the fraction of true positive test results from the population of PCR-positive samples. Specificity is defined as the fraction of true negative test results from the samples that were PCR-negative for the tested serotype. The optimal cutoff values, in which the sum of sensitivity and specificity was maximized, were: 0.32 for DENV-1, 0.25 for DENV-2, 0.47 for DENV-3, 0.45 for DENV-4, and 0.39 for pan-DENV. Based on the optimal cutoff, sensitivity and specificity values, respectively, across the four serotypes and PAN were calculated: 79.17% (38/48) and 68.06% (98/144) for DENV-1, 80.41% (78/97) and 88.08% (266/302) for DENV-2, 100% (45/45) and 94.07% (127/135) for DENV-3, 79.67% (98/123) and 77.75% (290/373) for DENV-4, and 79.66% (325/408) and 84.45% (1037/1228) for Pan DENV (Table 1, Fig 1). The AUC of the receiver operator characteristic (ROC) graph ranges from 0 to 1 and increases with test performance. The AUC was 0.74 for DENV-1, 0.88 for DENV-2, 0.99 for DENV-3, 0.82 for DENV-4, and 0.88 for PAN DENV (Table 1, Fig 1). Based on these measures of test performance, the DENV NS1 ELISA demonstrates a high sensitivity and specificity for characterizing serotype 1–4 and PAN dengue.

**Table 1:** Performance analysis of serotype DENV NS1 ELISA Test. Area Under Curve (AUC) of ROC Curve, 95% Confidence Interval (95% CI), Optimal Cutoff, Sensitivity (%), Specificity (%), and sample counts as represented by country and aggregate data.

		DENV-1	DENV-2	DENV-3	DENV-4	PAN-DENV
**Aggregate (n = 1630)**	AUC	0.74	0.88	0.99	0.82	0.85
	95% CI	0.66–0.83	0.84–0.92	0.98–1.00	0.78–0.86	0.83–0.87
	Cutoff	> 0.32	> 0.25	> 0.47	> 0.45	> 0.39
	Sensitivity	79.17	80.41	100	79.67	79.66
	Specificity	68.06	88.08	94.07	77.75	84.45
	N Total Positive	48	97	45	123	408
	N Total Negative	144	302	135	373	1228
**Honduras** **(n = 172)**	AUC	_	0.93	_	_	0.86
	95% CI	_	0.85–1.00	_	_	0.80 to 0.92
	Cutoff	_	> 0.09	_	_	> 0.16
	Sensitivity	_	88.89	_	_	62.79
	Specificity	_	94.44	_	_	93.02
	N Total Positive	0	18	0	0	43
	N Total Negative	0	54	0	0	129
**India (n = 260)**	AUC	_	0.90	_	_	0.92
	95% CI	_	0.84–0.95	_	_	0.88 to 0.95
	Cutoff	_	> 0.21	_	_	> 0.18
	Sensitivity	_	74	_	_	80
	Specificity	_	95.03	_	_	88.72
	N Total Positive	0	50	0	0	65
	N Total Negative	0	161	0	0	195
**Brazil** **(n = 1204)**	AUC	0.74	0.91	0.99	0.82	0.88
	95% CI	0.66–0.83	0.86–0.97	0.98–1.00	0.78–0.86	0.86–0.90
	Cutoff	> 0.32	> 0.48	> 0.47	> 0.45	> 0.39
	Sensitivity	79.17	96.55	100	79.67	86
	Specificity	68.06	78.16	94.07	77.75	79.65
	N Total Positive	48	29	45	123	300
	N Total Negative	144	87	135	373	904

### Performance of DENV NS1-ELISA for serotype and PAN diagnosis by Country [Table 1 and Figs 1 and 2A–2F]

**Fig 2:**
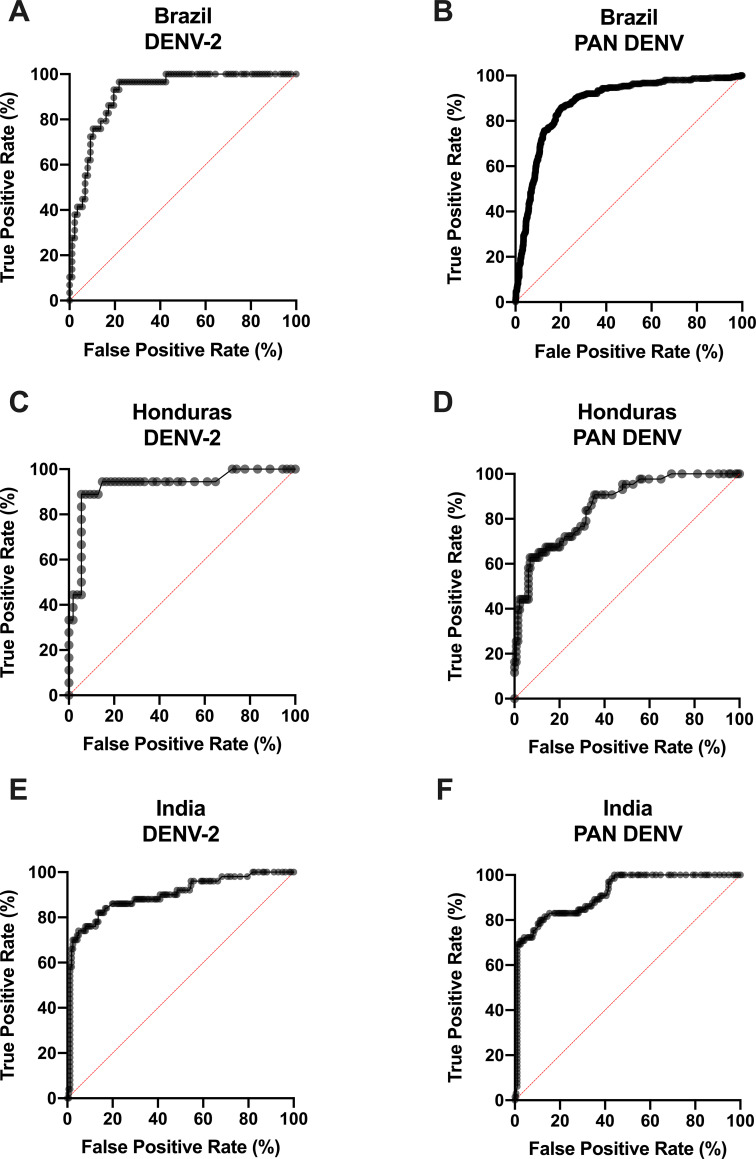
Performance of serotype DENV NS1 ELISA test by country. Receiver Operating Characteristic (ROC) Curve analysis for DENV-2 samples in Brazil (A), PAN DENV samples in Brazil (B), DENV-2 samples in Honduras (C), PAN DENV samples in Honduras, DENV-2 samples in India (E), PAN DENV samples in India (F). Performance is demonstrated in terms of True Positive Rate (%) versus False Positive Rate (%). Performance of Brazil DENV1, DENV3, and DENV4 tests are presented as aggregate data in Fig 1.

To understand the performance of the serotype and PAN NS1-ELISA geographically, ROCs were generated for Brazil, Honduras, and India individually. The optimal cutoff values for Absorbance OD 450 nm for DENV-2 and pan-DENV, respectively, were: 0.48 and 0.39 for Brazil, 0.09 and 0.16 for Honduras, and 0.21 and 0.18 for India (Table 1). DENV-1, DENV-3, and DENV-4 samples were tested in Brazil, and the ROCs were represented as aggregated data as these serotypes could only be collected in Brazil (Fig 1). According to the cutoff values, the sensitivity and specificity, respectively, were: Brazil demonstrated a performance of 96.55% (28/29) and 78.16% (68/87) for DENV-2 and 86% (258/300) and 79.65% (720/904) for pan-DENV detection. Honduras demonstrated a sensitivity and specificity of 88.89% (16/18) and 94.44% (51/54) respectively for DENV-2, and 62.79% (27/43) and 93.02% (120/129) for pan-DENV. India demonstrated a sensitivity and specificity, respectively, of 74.00% (37/74) and 95.03% (153/161) for DENV-2, and 80.00% (52/65) and 88.72% (173/195) for pan-DENV (Table 1, Fig 2). The AUCs for overall test performance were as follows: 0.74 (DENV-1), 0.91 (DENV-2), 0.99 (DENV-3), 0.82 (DENV-4), and 0.85 (Pan DENV) for Brazil. For Honduras, the AUCs were 0.93 (DENV-2) and 0.86 (Pan DENV), and for India 0.90 (DENV-2) and 0.92 (Pan DENV) (Table 1, Fig 2). Taken together, the serotype and pan-DENV NS1 ELISA demonstrated high sensitivity and specificity in aggregated analysis, as well as by geo specific location analysis.

## Discussion

Despite widespread circulation and ongoing outbreaks of DENV serotypes, there currently exists no high-throughput DENV serotype screening method for clinical use. PCR serotyping remains a costly and time-intensive diagnostic technique, requiring experienced personnel to prepare samples for nucleic acid extraction. The rapid degradation of RNA and the risk of amplicon contamination which can lead to false positives further render PCR impractical. Despite also requiring machinery and personnel, ELISAs offer a quicker and less expensive alternative for serotype diagnosis in both limited and abundant resource settings while overcoming the limitations of relying on RNA. In this study, we present a highly specific and sensitive NS1-based ELISA for determining the serotype and presence of DENV infection based upon our previous generation of antibodies [33].

Previous methods for serotype identification using an NS1 ELISA have been reported. Lebani et al. utilized phage display to isolate serotype-specific NS1 DENV antibodies for an ELISA. Along with using costly machinery to detect fluorescent microsphere beads in their assay, they used human serum spiked with virus as opposed to infected patient samples in their assays [34]. Röltgen and Lai developed a serotype NS1 DENV ELISA but they each used approximately 60 positive serum samples, of which all or a majority originated from Asia [27, 28].

This study is significant because it overcomes several of the challenges faced in previous attempts. Our study demonstrates a robust antibody screening which led to the development of an NS1-based ELISA for DENV serotyping. This assay presents high sensitivity and specificity, utilizing the most expansive multinational cohort to date. Approximately half of the patient cohort is from Latin America, a geographic location underrepresented in previous studies [27, 28]. In 2015, 2.35 million DENV infection cases were reported in the Americas alone [4]. Latin America, especially Brazil and Honduras, bear a tremendous burden of dengue, along with chikungunya and Zika–all of which are spread by the same vector and share the same nonspecific clinical symptoms. Our previous work demonstrated that our DENV antibodies do not cross-react with ZIKV [33]. Our DENV-1-4 ELISA is a step towards differential diagnosis and the development of a more nuanced surveillance of circulating diseases. Because the NS1 antigen is optimally detectable within the first 5 days of fever, this ELISA offers an opportunity for early detection of disease and serotyping, particularly in comparison to IgG/IgM-based assays. The results of this study are significant due to the high overall sensitivity and specificity, of which data for DENV2 and pan-DENV use samples from three different countries. Sensitivities across all DENV serotypes and pan-DENV for our ELISA were above 79.17%, with the sensitivity of DENV-2 and DENV-3 serotype being 80.41% and 100% respectively. These sensitivities compare very well to previous attempts to develop an NS1-DENV ELISA for serotype diagnosis [27, 28].

Because NS1 is secreted into the blood during the acute phase of infection, optimal detection and diagnosis is limited to the first 5 days of fever post-infection. Though this promotes early diagnosis and preparedness, the sensitivity values were likely affected by sample collection taking place in the latter end of the optimal diagnosis window. The consequential lower viremia in samples likely affected cutoff and sensitivity values in Honduras, for instance. Ideally, patient samples are stored in -80˚C until diagnostic use to prevent protein degradation. In Honduras, temporary temperature fluctuations of stored samples possibly led to protein degradation, affecting sensitivity values. However, despite the discrepancies in sensitivity and cutoff values, the AUC for Honduras remained high at 0.9275 (DENV-1) and 0.8596 (PAN), demonstrating high overall sensitivity and specificity. More broadly, the AUC of all countries and serotypes ranged from 0.7418 to 0.988, further demonstrating overall strong and relatively consistent test performance. Future studies will involve a method for documenting the specific day of fever to further investigate the relationship between viremia, day of fever and NS1 detection by ELISA. Moreover, we intend to further test this ELISA using serotypes in multiple countries to gather even greater globally representative data of diagnostic performance.

Taken together, this specific and sensitive DENV NS1-ELISA for serotype diagnosis offers multiple opportunities to develop more targeted treatment and prevention of widespread DENV. The current economic burden of DENV was estimated to be $8.9 billion in 2013, before several more outbreaks occurred in 2015 and 2016 [35]. The progression to severe dengue disease (SDD) and death, as well as the costs that hospitals and patients incur are often attributed to late diagnosis. When paired with disease awareness, this NS1-based assay enables a platform in which disease can be detected early, supportive treatment may be administered, and further transmission may be prevented. Several reports have correlated DENV serotype with severity of symptoms [11–13, 16, 17, 28, 36]. In Brazil, for instance, it was previously reported in a study of 452 DENV-infected patients, that DENV-1 patients presented more spontaneous bleeding, intense abdominal pain, and neurological symptoms when compared to the circulating DENV-4 serotype [13]. Along with correlations between serotype and severity, heterologous secondary infection is associated with increased symptom severity due to antibody-dependent enhancement. Mehta et al reported thrombocytopenia, hemorrhagic manifestations and atypical presentations were common amongst patients infected with DENV-2 following a previous DENV-3 infection [36]. Understanding the serotype history of patients can be instrumental in necessary triaging of patient care in hospitals.

This ELISA method is also significant for epidemiological measures to control and prevent DENV spread. DENV serotyping is now even more critical with the finding that the first and presently only available DENV vaccine Dengvaxia increases the risk of severe illness amongst seronegative individuals [37]. The World Health Organization has recommended that patients be screened for their serostatus prior to receiving the vaccine [38]. Our serotype NS1 ELISA offers a high-throughput screening method for patients suffering from fever, which can provide crucial patient information prior to dengue vaccination to enable disease control.

The methods and results described in this study offer insights to further develop our previously reported rapid lateral flow dengue serotype diagnostic. Ideally, rapid diagnostics and ELISA methods may be combined to provide patients with point-of-care diagnostics that may be confirmed in a clinical laboratory. In summary, we present a highly specific and sensitive NS1-ELISA for serotyping DENV using infected samples from three countries. Understanding the serotype of DENV-infected patients may be used in disease surveillance systems to equip healthcare stakeholders and patients with tools to control transmission, administer necessary care, and prevent further global outbreaks.

## Supporting information

S1 Checklist STROBE checklist. (DOC)

S1 Table Limits of detection of antibody pairs to detect DENV Serotype. The limits of detection (LoD) and dissociation constant (K_d_) were calculated for each antibody pair for the detection of its respective dengue serotype using ELISA (a) and dipstick (b) formats.
(DOCX)

S1 Fig Monoclonal antibody clones were generated from four DENV-immunized mice: HM7729 (A-C), HM7732 (D-F), MA724 (G-J), and MA725 (K-M). The OD_450_ when antibodies were treated with DENV 1, 2, 3, or 4 NS1 is represented as fold above background.
(TIFF)

S2 Fig Monoclonal antibody clones were generated from four DENV-immunized mice: HM7729 (A), HM7732 (B), MA724 (C), and MA725 (D). X values represent OD_450_ fold above background for one DENV serotype NS1, and Y values represent fold value for three remaining serotypes.
(TIFF)

S3 Fig Limits of Detection of Antibody Pairs to detect DENV Serotype. Limits of Detection (LoD) using increasing concentrations of DENV NS1 were using ELISA or dipstick formats for antibody combinations 271 and 912 **(A)**, 323; 243 **(B)**, 243; 164 **(C)**, 55; 411 **(D)**, 55; 626 **(E)**, 323; 243, 271, 411, 626 **(F).**
(TIFF)
