# Supplementary material for: Serotype-specific detection of dengue viruses in a nonstructural protein 1-based enzyme-linked immunosorbent assay validated with a multi-national cohort
Source: PLoS Negl Trop Dis. 2020 Jun 24;14(6):e0008203. doi: 10.1371/journal.pntd.0008203 (PMC7351204; doi:10.1371/journal.pntd.0008203)
Supplement: S1 Table — The limits of detection (LoD) and dissociation constant (Kd) were calculated for each antibody pair for the detection of its respective dengue serotype using ELISA (a) and dipstick (b) formats. (DOCX) [file pntd.0008203.s002.docx]

|  | **DENV1^a^** | **DENV2 (Brazil, Honduras)^a^** | **DENV2 (India)^a^** | **DENV3^b^** | **DENV4^a^** | **PAN DENV^a^** |
| --- | --- | --- | --- | --- | --- | --- |
| **Antibody Pair** | 271; 912 | 323; 243 | 243; 164 | 55; 411 | 55; 626 | 323; 243, 271, 411, 626 |
| **LoD (ng/mL)** | 128.53 | 33.96 | 69.74 | 270.20 | 58.09 | 53.08 |
| **Kd (ng/mL)** | 385.59 | 101.89 | 209.22 | 694.79 | 174.28 | 159.25 |

SUPPORTING INFORMATION

Supplementary Table 1: Limits of Detection of Antibody Pairs to detect DENV Serotype.
