# Supplementary figures and images for: Serotype-specific detection of dengue viruses in a nonstructural protein 1-based enzyme-linked immunosorbent assay validated with a multi-national cohort
Source: PLoS Negl Trop Dis. 2020 Jun 24;14(6):e0008203. doi: 10.1371/journal.pntd.0008203 (PMC7351204; doi:10.1371/journal.pntd.0008203)

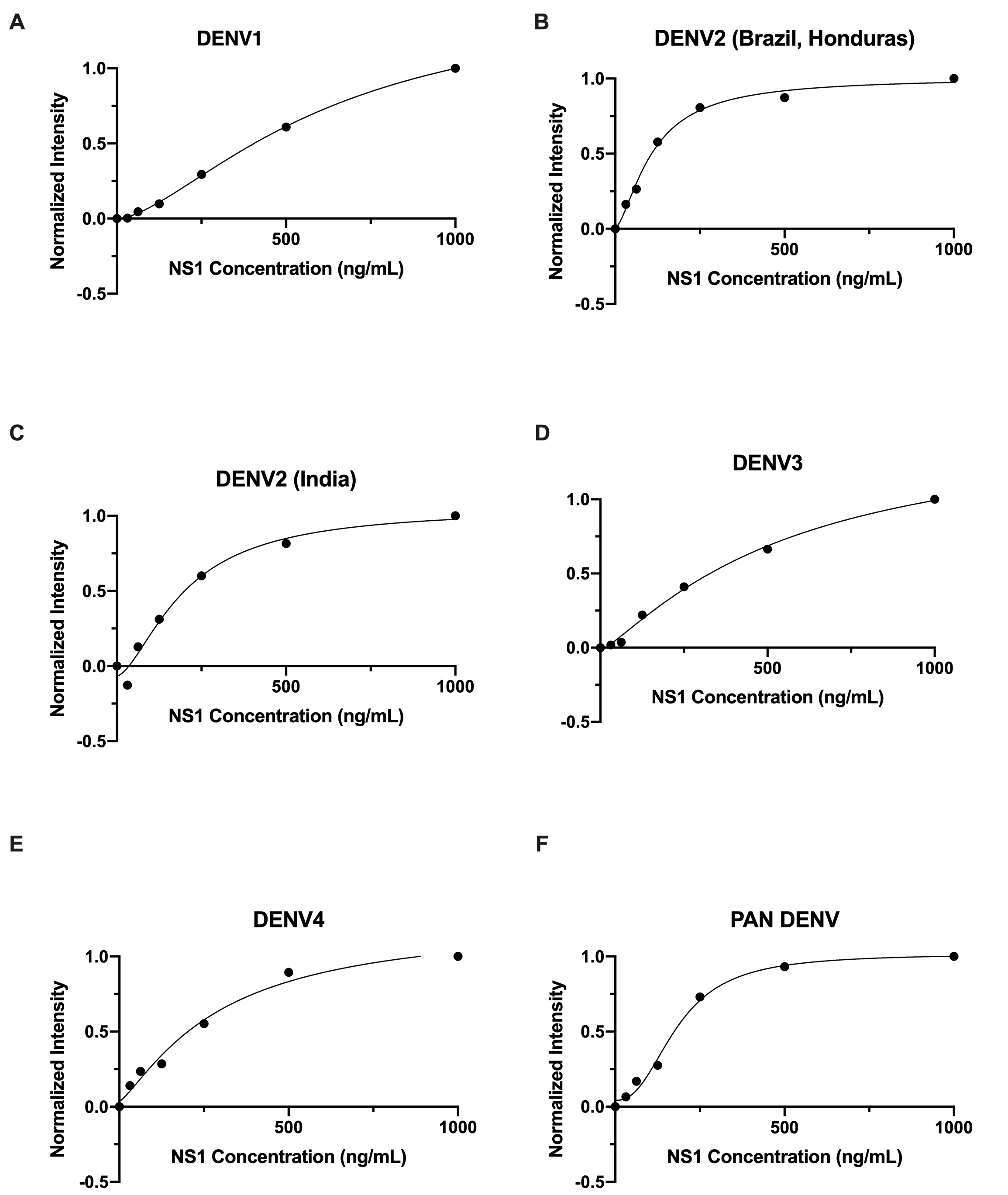

Supplement: S3 Fig — Limits of Detection (LoD) using increasing concentrations of DENV NS1 were using ELISA or dipstick formats for antibody combinations 271 and 912 (A), 323; 243 (B), 243; 164 (C), 55; 411 (D), 55; 626 (E), 323; 243, 271, 411, 626 (F). (TIFF) [file pntd.0008203.s005.tiff]
